# Peptide Regulation of Gene Expression: A Systematic Review

**DOI:** 10.3390/molecules26227053

**Published:** 2021-11-22

**Authors:** Vladimir Khatskelevich Khavinson, Irina Grigor’evna Popovich, Natalia Sergeevna Linkova, Ekaterina Sergeevna Mironova, Anastasiia Romanovna Ilina

**Affiliations:** 1 Department of Biogerontology, Saint Petersburg Institute of Bioregulation and Gerontology, 197110 Saint Petersburg, Russia; vladimir@khavinson.ru (V.K.K.); irina_popovich@inbox.ru (I.G.P.); katrine1994@mail.ru (E.S.M.); ilinaanastasiar@gmail.com (A.R.I.); 2 Group of Peptide Regulation of Aging, Pavlov Institute of Physiology of the Russian Academy of Sciences, 199004 Saint Petersburg, Russia

**Keywords:** short peptides, DNA–peptide interactions, histones, epigenetics, peptide drugs

## Abstract

Peptides are characterized by their wide range of biological activity: they regulate functions of the endocrine, nervous, and immune systems. The mechanism of such action of peptides involves their ability to regulate gene expression and protein synthesis in plants, microorganisms, insects, birds, rodents, primates, and humans. Short peptides, consisting of 2–7 amino acid residues, can penetrate into the nuclei and nucleoli of cells and interact with the nucleosome, the histone proteins, and both single- and double-stranded DNA. DNA–peptide interactions, including sequence recognition in gene promoters, are important for template-directed synthetic reactions, replication, transcription, and reparation. Peptides can regulate the status of DNA methylation, which is an epigenetic mechanism for the activation or repression of genes in both the normal condition, as well as in cases of pathology and senescence. In this context, one can assume that short peptides were evolutionarily among the first signaling molecules that regulated the reactions of template-directed syntheses. This situation enhances the prospects of developing effective and safe immunoregulatory, neuroprotective, antimicrobial, antiviral, and other drugs based on short peptides.

## 1. Introduction

Peptides are molecules that contain 2–100 amino acid residues bonded by amide (peptide) bonds. Peptides can be considered either as polypeptides or oligopeptides depending on the number of amino acid residues present. Here, polypeptides are assumed to contain up to 100 amino acid residues per molecule, whereas oligopeptides (short peptides) only contain up to 10. Macromolecules containing over 100 amino acid residues are called proteins. Note, however, that according to the International Union of Pure and Applied Chemistry (IUPAC) classification, ‘short’ peptides consist of 10–20 amino acid residues, whereas ‘polypeptides’ include 20 or more amino acid residues [1,2]. Meanwhile, another classification considers ‘short’ peptides to include compounds of up to 40 amino acid residues [3]. Moreover, there is a group of ultrashort peptides distinguished, according to some literature reports, as consisting of only 2–4 amino acid residues [4], while, according to another, of 3–7 amino acids [5]. 

In general, regardless of the number of amino acid residues in their composition, peptides support many key processes in the body due to their antioxidant, antimicrobial, antibacterial, anti-inflammatory, anticarcinogenic, antitumor, and immunoregulatory characteristics [6,7,8].

Peptides perform various biological functions: they regulate the functions of the endocrine, nervous, and immune systems. The activities of peptides are characterized by their wide range of biological properties, including their regulation of cell differentiation, apoptosis, and proliferation. Thus, the study of the mechanisms of the physiological activity of peptides is of great interest for researchers working in the sphere of molecular biology, pharmacology, and medicine [9].

**Figure 1:**
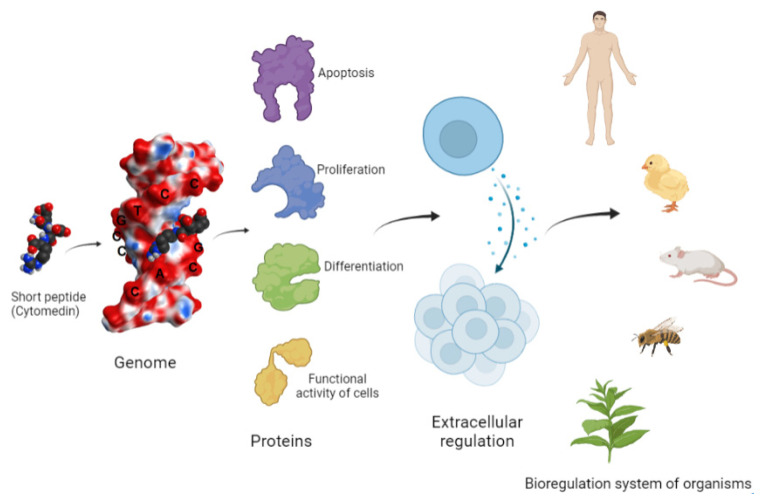
Bioregulation system of a multicellular organism (according to Morozov and Khavinson, 1983, with modifications) [11].

The isolation of polypeptide extracts from various animal organs, and the further construction and synthesis of short peptides with a length of 2–4 amino acid residues, was an important stage in the study of the biological activity of peptides [8,10]. The authors revealed the so-called cytomedines (from the Greek word “κύτος”—”cell” and the Latin word “mediator”—”intermediary”), the activities of which are characterized by a wide range of biological properties, including those in intercellular signaling [11,12]. A scheme was proposed, according to which the information entering the body, as well as the response signal, are both controlled by the biological regulation system (Figure 1). The main task of this biological regulation system is to regulate the genome and ensure the operation of protective functions (immunity, reparative, and adaptive processes), which have regulatory mechanisms in common. It was assumed that, when penetrating into a cell, the regulatory peptides interacted with the genome and thus controlled its functional activity.

Studies of the biological activity of cytomedines obtained from the tissues of various organs have demonstrated the specificity of their action in relation to the cell populations that are the origins of them. The results indicated the participation of cytomedines in the regulation of cell differentiation and proliferation, as well as their ability to change the functional activity of the genome in different phases of the cell cycle [11,12,13].

Further experimental studies in vitro and in vivo established the extensive biological activity of polypeptides and short peptides, in immunomodulatory, anticarcinogenic, and geroprotective activity [8,14]. Such identified biological effects of cytomedines have spawned many studies of their mechanisms of action. Numerous studies have demonstrated a specific DNA–peptide interaction [15,16,17,18,19,20,21,22]. Peptides EDR, AEDG, AEDL, KEDW, AEDR, and KEDG can bind to histone proteins H1, H2b, H3, and H4, increasing the transcription availability of gene promoter zones [15]. Peptides KE, AEDG, and AEDL stimulate the expression of the CLE, KNOX1, and GFR gene families involved in the Nicotiana tabacum plant cell differentiation [16]. Peptides KE, AED, KED, and AEDG regulate the expression of genes for neurogenesis (nestin, βtubulinIII, doublecortin, GAP43, SUMO), ageing (p16, p21), functional activity (IFG-1, FOXO1, TERT, TNKS2, NFkB), and circadian rhythms (Clock, Csnke1, Cry2) in stem cells and human blood lymphocytes through specific DNA–peptide interactions and/or binding to histones H1/3 and H1/6 [17,18,19,20,21,22].

This research has led to the conclusion that the biological effects of peptides are based on their ability to regulate gene expression and protein synthesis through such DNA–peptide interactions [23,24]. 

It should be noted that the first works on the possible DNA–peptide interaction conducted between 1975 and 1979 were mainly related only to the specifics of the action of low-molecular-weight peptides obtained from the thymus gland [25,26].

However, the authors did not associate the physical and chemical properties of the peptides with their potential biological function.

This review is dedicated to studying the inter-relation of the biological function of peptides and their ability to regulate the expression of genes by means of their binding to the DNA. There are very few studies in this area as compared, for example, to the considerable data available on the DNA methylation and/or histone acetylation, peptide binding with transcription factors, etc. [27]. However, studies of the patterns of DNA–peptide interactions identified in vitro help to characterize the specificity of action of short peptides. Furthermore, investigation of such a mechanism of peptide action may become the basis for the development of drugs for various disease treatments. Consequently, the objective of this review is to provide an overview of the information available on the role of DNA–peptide interactions within the regulation of gene expression, protein synthesis, and physiological functions.

## 2. Biological Effects of Peptides 

Modern ideas about peptide compounds indicate that peptides obtained in a number of different ways (hydrolysis, extraction, and/or synthesis) have a wide range of biological effects: immunomodulatory, neuroprotective, antimicrobial, antiviral, anticarcinogenic, etc.

There are several comprehensive reviews on marine-derived peptides. Their authors describe the antihypertensive, antioxidant, antimicrobial, neuroprotective, and geroprotective properties of these peptides in detail, but they also emphasize the difficulties they faced in extraction and purification of marine-derived peptides, which consequently may serve as an obstacle for drug design [6,7,28,29]. 

Maestri et al., in their meta-analysis, provided an extensive overview of the contributions related to 807 biologically active peptides from food products of animal origin (milk, meat, eggs, and seafood). It is significant that the authors succeeded in correlating peptide structure with activity (antihypertensive, antioxidant, immunomodulatory, antimicrobial, hypolipidemic, antithrombotic, and opioid) and stability in vivo [30]. It should be stressed, however, that present-day ideas on the biological activity of peptides also include neuroprotective and other effects that were not described therein.

A wide range of the biological activities of extracts obtained from cattle organs, as well as of some synthetic peptides, have been studied [8]. It was demonstrated that peptide extracts isolated from the tissues of various organs have pronounced geroprotective, anticarcinogenic, immunomodulatory, and neuroprotective properties, including the ability to regulate the functions of the endocrine system [14,31]. When studying their composition, the main components of the extracts were identified as di- and tetra-peptides, both having similar biological effects [8]. 

Thus, many years of studying the biological characteristics of peptides have made it possible to draw conclusions about the ability of such compounds to regulate physiological processes in both normal and pathological conditions. 

## 3. Peptide Regulation of Gene Expression and Protein Synthesis 

Gene expression mediates cell processes from differentiation, functional activity, and apoptosis [32]. Understanding the mechanisms of regulation of gene expression makes it possible to determine how to correct the vital activity of cells that have been impacted by a range of pathologies. Short peptides with a molecular weight of up to 3 kDa are considered to be especially active in the regulation of gene expression, having the ability to penetrate the cytoplasmic and nuclear membranes of cells [33] and influence the expression of specific genes [23,34,35]. AEDG and KE peptides were found to regulate the expression of 98 and 36 genes, respectively [23,34]. It was revealed that the AEDG and AEDP tetrapeptides activate the differentiation of pluripotent cells towards epidermis, mesenchymal, and nervous tissue. Peptides KE, AED, KED, AEDG, and AAAAEKAAAAEKAAAAEK are activators of neuronal differentiation. The AEDL peptide stimulates the lung cells’ differentiation, while the KEDW peptide stimulates the maturation of various types of human pancreatic cells. Peptides that activate immunogenic differentiation are KE, DS, (Nα- (γ-E) -E), K (H-E-OH) -OH, AED, KED, EDA, and KEDG. Regulators of osteogenic differentiation include IRW, GRGDS, and YCWSQYLCY peptides. In each case, a peptide activates the expression of genes encoding proteins, characteristic of the phenotype of the corresponding subpopulation of cells of a given tissue [35]. This property of peptides is of the utmost importance as it indicates the significant role of the peptide regulation of gene expression in such biologically important processes as cell differentiation, functional activity, senescence, apoptosis, immunogenesis, and neurogenesis. 

### 3.1. Peptide Regulation of Cell Differentiation

Cell differentiation is one of the main physiological mechanisms maintaining the functional activity and homeostasis of organs and tissues. In the case of senescence and in various pathologies, one can often see a violation of cell differentiation. Thus, identification of substances that induce differentiation is a hot topic in biogerontology and regenerative medicine. Short peptides are considered as one such group of biologically active molecules. 

Self-assembling peptides (SAPs), referred to as modified peptides, are important in regulating differentiation. These short peptides contain amino acid residues modified by covalent bonds that result in them organizing themselves into nanofiber structures. The peptide IKVAV is a member of the SAP group, and has been shown to activate the neurogenic differentiation of stem cells [36]. The peptides KLDL and RADA are stimulants of osteogenic and chondrogenic differentiation. RADA, in combination with the Notch-1 factor ligand (Jagged1), activates differentiation of heart cell progenitors. This is seen in the stimulation of the expression of the genes for the following transcription factors: Nkx2.5, Hey1, MEF2C, and GATA4 [37]. It has been suggested that the ability of SAPs to induce the directed differentiation of stem cells opens up their potential for consideration in the development of drugs that could be effective in cardiovascular pathologies and neurodegenerative diseases (Alzheimer’s and Parkinson’s) [36]. The tripeptide RGD has been shown to stimulate the osteogenic differentiation of mesenchymal stem cells [38]. In contrast, the short, cyclic peptides A–I F and H I inhibit the ability of osteoclasts to differentiate [39]. 

The direction of induction of cell differentiation depends on the structure and concentration of the peptide(s) controlling it. The range of effective concentrations of short peptides regulating cell differentiation has generally been found to be from 2 to 200 ng/mL, but depends on the structure of the peptide, the characteristics of the cell culture being studied, and other aspects of the experimental design [34].

The peptides AEDG and AEDP invoke differentiation of pluripotent cells of the epidermis, mesenchyme, and nervous tissue [17,20,34]. Peptides KE, AED, KED, AEDG [34], and AAAAEKAAAAEKAAAAEK [40] activate neuronal differentiation. It has been demonstrated that the AEDL peptide induces the differentiation of lung cells by regulating the expression of the *NKX2-1*, *SCGB1A1*, *SCGB3A2*, *FOXA1*, and *FOXA2* genes [41]. Another study showed that the peptide KEDW increases the expression of the genes *PDX1*, *NGN3*, *MNX1*, *PAX6*, *FOXA2*, *NKX2-2*, *NKX6.1*, *HOXA3*, and *PAX4* that are important for maintaining the functional activity of pancreatic endocrine cells [42]. It has been established that directed differentiation of immune cells is stimulated by the peptides KE, DS, (Nα- (γ-E) -E), K (H-E-OH) -OH, AED, KED, EDA, and KEDG [34]. Meanwhile, IRW [43], GRGDS [44,45], and YCWSQYLCY [46] activate the osteogenic differentiation of stem cells. 

Furthermore, the KE, AEDL, and AEDG peptides can induce the differentiation of plant cells too. In particular, AEDG and AEDL enhance culture growth and can stimulate the formation and growth of leaves in regenerated plants of tobacco callus cultures (*Nicotiana tabacum*). As the regulatory activity of short peptides is manifested at low concentrations, their action is to some extent similar to that of phytohormones, has a signaling character, and is epigenetic in nature. The peptides that have been studied so far modulate gene expression in tobacco cells, including the genes responsible for tissue development and cell differentiation. These peptides modulate the expression of the CLE family of genes that code known endogenous regulatory peptides, the KNOX1 genes (transcription factor genes), and the GRF family of genes (growth regulatory factors) that code for the corresponding DNA-binding proteins, such as topoisomerases, nucleases, etc. [16,24].

Studies in the field of stem cell-directed differentiation by means of peptide regulation are of particular importance for the development of innovative approaches in molecular medicine and cell therapy. Such peptides may become useful additional tools to stimulate cell differentiation, tissue regeneration, and cytoprotection in the treatment of various human diseases, while other uses for peptides will likely be found in experimental botanics, molecular plant biology, biotechnology, and practical agronomy.

### 3.2. Peptide Regulation of the Functional Activity of Cells 

The functional activity of cells underlies the maintenance of the body’s homeostasis but becomes impaired during disease and senescence. The main functions of eukaryotic cells include maintaining a constant intracellular electrolyte composition and the concentration of nutrients, including glucose, structuring the cytoskeleton, endo- and exo-cytosis, response to extracellular signals that determine viability, migration, proliferation, differentiation, and bioelectrogenesis [47]. Short peptides are signaling messengers that regulate such principal cell functions [48]. 

It has been demonstrated that, in the case of metabolic stress (lack of glucose), the peptide MOTS-c encoded by the mitochondrial genome transfers into the cell nucleus and activates the expression of nuclear antioxidant response genes. Thus, the MOTS-c peptide regulates cell homeostasis by linking the mitochondrial genome with the nucleus [49]. Another peptide that regulates metabolism is the gastrin-releasing peptide. This peptide activates proglucagon gene expression in STC-1-line enteroendocrine cells [50]. 

The AEDG peptide regulates expression of the circadian rhythm genes *Clock*, *Csnk1e*, and *Cry2* in the leukocytes and blood lymphocytes of people with suppressed melatonin-producing pineal gland function [19]. It was also previously found that AEDG restores the melatonin-forming function of the pineal gland that normally decreases during senescence, and that it both increases the lifetime and inhibits senescence of reproductive functions in experimental animals [51]. It is possible that AEDG regulates the expression of circadian rhythm genes not only in blood cells, but also in other organs and tissues, which would explain its geroprotective effect and influence on the functions of the organs in the neuro-immuno-endocrine systems. 

The hexapetide HAV (Ac-SHAVSS-NH2) regulates the expression of genes, the products of which are involved in E-cadherin synthesis and ensure adhesion of the intestinal mucosa cells (Caco-2 line). A change in expression of these genes under the influence of HAV is followed by a decrease in E-cadherin synthesis by 20% [52]. 

It has been found that AEDL is involved in regulation of the functional activity of the bronchial epithelium. This tetrapeptide activates the expression of the *MUC4*, *MUC5AC*, and *SFTPA1* genes, a decrease in expression of which correlates with the development of bronchopulmonary pathology [41]. 

The ability of short peptides to regulate the functional activity of brain cells has also been identified. In the case of cerebral ischemia in rats, the peptides MEHFPGP (an analog of the N-end fragment of the adrenocorticotropic hormone) and PGP demonstrated neuroprotective properties by regulating the expression of the vascular endothelium growth factor mRNA genes *Vegf-a*, *Vegf-b*, *Vegf -c*, *Vegf-d*, and *Plgf* that promote regeneration of the organum vasculosa [53,54]. Under real-time PCR, MEHFPGP, as well as the peptide drug Semax (the ethyl ester of N-phenylacetyl-L-prolylglycine), have been shown to increase the expression of the mRNA neurotrophin genes: for the nerve growth factor (NGF) and brain neurotrophic factor (BDNF), in different areas of the rat brain [55]. Furthermore, Semax also regulated gene expression and synthesis of the stress-induced SAPK/JNK46/54 and pERK1/2 kinases, activation of which is associated with the development of neurodegenerative diseases [56]. The tripeptide PGP increased the expression of the mRNA from genes that coded for neutrophines and their *TrkA*, *TrkB*, *TrkC*, and *p75* receptors in the frontal lobes of the brain, hippocampus, and cerebellum of rats under cerebral ischemia [53]. Moreover, MEHFPGP normalized the expression of the *c-Fos* gene and the synthesis of the protein encoded by this gene in the paraventricular hypothalamus and medial septum of the brains of rats predisposed to emotional stress [57]. The heptapeptide TKPRPGP regulated the mRNA expression of 45 genes involved in the neurotransmission processes (the main subunit of the GABA receptor, ion channel proteins, dopamine and serotonin receptors, etc.) in the frontal cortex of the rat brain [58,59]. 

It is significant that the KED vasoprotective peptide [60], which has a neuroprotective effect in models of Alzheimer’s disease in mice in vitro and in vivo [61,62], regulated the mRNA involved in expression of the cell senescence and apoptosis genes (*p16*, *p21*), of neurogenesis (*NES*, *GAP43*), and of other genes involved in the pathogenesis of Alzheimer’s disease (*SUMO1*, *APOE*, *IGF1*) [22]. It should be noted that as such short peptides can regulate the expression of genes responsible for functions of the cell populations of the brain, this indicates promising prospects for using short peptides for neuroprotection.

Another area involving peptide regulation of gene expression is the regulation of the functional activity of cells of the immune system. It was found that the immunomodulatory peptide TKPRPGP alters the mRNA expression of 34 genes for chemokines, cytokines, and their receptors in the mouse spleen. In particular, the greatest changes under the influence of the peptide were observed in the expression of the mRNA of the *Bcl6* gene, which is of major importance in the elaboration and development of the immune system [63,64]. The genome-wide RatRef-12 Expression BeadChip (Illumina, San Diego, CA, USA) has been used to study changes in genome-wide expression caused by MEHFPGP in tissues of the rat cerebral cortex during focal ischemia. It was shown that this short peptide changes the expression of mRNA for 96 genes associated with the number and mobility of immune cells, the functional activity of chemokines and immunoglobulins, as well as those involved in such processes as the development and migration of endothelial tissue cells, the migration of smooth muscle cells, hematopoiesis, and vasculogenesis [65,66]. Thus, peptide regulation of the functional activity of cells of the immune system could form the foundation of immunoprotection with short peptides. Analysis of the above studies indicates that short peptides have common immuno- and neuro-protective properties. This could provide a basis for the complex therapy of neurodegenerative conditions. 

A previously mentioned study that used DNA microarray technology established the ability of the peptides EW, KE, and AEDG to regulate the expression of a wide range of genes [23]. A trend towards investigating the ability of peptides to regulate such biological processes as energy homeostasis, inflammation, apoptosis, cell stress, etc., was found based on the GeneCards database. Moreover, links of the listed genes with the development of various pathologies, including neuropathies, immunodeficiencies, and cancer (Table 1, Table 2 and Table 3), were revealed.

Thus, the literature sources demonstrate the results of numerous studies proving the ability of short peptides to regulate the expression of genes associated with various types of the cell functional activity and a wide range of biological processes. The association of genes, the activity of which is regulated by short peptides, with the development of pathological processes, unveils the prospect of using short peptides as biologically active compounds to regulate physiological processes, as well as in developing drugs for the treatment of various pathologies.

### 3.3. Peptide Regulation of Senescence and Apoptosis

Taking into account the forecasted increase in life expectancy, it is now important to develop strategies to maintain active longevity. Short peptides form one of the promising groups of geroprotectors that could accomplish this. Cell senescence is a process that results in an irreversible arrest of the cell cycle. In the case of senescence, cells develop distinctive metabolic and signaling features, collectively referred to as the senescence-related secretory phenotype (SASP). Onset of the SASP is typical for several age-associated pathologies, including various malignant mass lesions. Peptides are one of the potential therapeutic agents for the suppression of SASP and for the prevention of the adverse effects of cell senescence [67].

For instance, mitochondrial peptides (MDPs) are important mitochondrial components that activate signaling pathways and modulate the expression of nuclear genes. MDPs are well-represented by the peptides humanin and MOTS-c. In the case of senescence, the synthesis of these peptides decreases, resulting in a loss of physiological function. Falling MDP levels are also associated with age-related diseases [68].

The FOXO4 transcription factor is known to support the viability of senescent cells by suppressing their response to apoptosis. Interfering with FOXO4 signaling may be a strategy to eliminate senescent cells and has potential as a treatment for age-related diseases. Studies of the chemical nature of peptides have demonstrated that protein domains containing natural L-peptides could sometimes be simulated by using D-amino acids in the reverse order [69]. Modification of peptides with this D-Retro Inverso (DRI) isoform can give new chemical properties to peptides, which in turn can improve their efficacy in vitro and in vivo [70]. In clinical trials, several DRI-modified peptides proved to be well-tolerated and therapeutically effective. For instance, a double-blind, randomized, placebo-controlled phase IIb study [71] and phase I study of the systemic treatment of solid tumors [72] demonstrated the advantage of DRI peptides in clinical therapy. It was found that a synthesized peptide in the DRI conformation called FOXO4-DRI disrupts the FOXO4 interaction with p53. In senescent cells, this causes p53 translocation from the nucleus that results in the development of apoptosis [73]. 

The AED and EDL peptides were found to regulate the expression of the p53 apoptosis marker in renal cell cultures during senescence [74]. Furthermore, the peptides AcSDKP, KED, AEDG, and AED slow down apoptosis and stimulate skin cell proliferation, as well as increasing the functional activity of skin fibroblasts and normalizing hemostasis of the intracellular matrix [75]. It has been found that the peptide AB-9 stimulated proliferative activity and differentiation of thymocytes in addition to suppressing apoptosis of thymus cells [76]. 

With an increase in the number of their divisions, mesenchymal stem cells enter a senescence stage that is characterized by a change in the typical morphology of their fusiform shape. Such cells become unsuitable for clinical use, where a large number of stem cells is required. AEDG and KED were found to reduce gene expression and the synthesis of the replicative senescence p16 and p21 proteins in stem cell cultures of the periodontal ligament and in human gingival mesenchymal stem cells [21].

It was also found that the short peptides AED, KED, and KE modulate the expression of the IGF1, FOXO1, TERT, TNKS2, and NFκB cell senescence genes in bone marrow mesenchymal stem cells (FetMSC line) in various models of cell senescence [18]. 

Thus, short peptides can regulate the functional activity of cells in the case of senescence. This makes it possible to use short peptides as regulators of regenerative processes, as well as for providing additional components in culture media that can support the functional state of cell material during replacement therapy for age-related diseases. 

## 4. Interaction of Short Peptides with DNA and Histone Proteins

Current literature explains peptide-modified regulation of gene expression by the interaction of peptides with DNA [23,24]. For example, in the above-mentioned review [30], the description of the mechanism of the biological effects of peptides only touches on their ability to regulate gene expression. At the same time, as it was mentioned earlier, gene expression may be regulated by different mechanisms, DNA methylation, and/or histone acetylation, as well as transcription factors [27]. In the following, the authors will explore the mechanisms of peptide interaction with DNA and histones to explain the specificity of peptides’ biological action. 

### 4.1. Short Peptides + DNA

It is important to note a number of experimental physical and chemical studies indicating the possibility of the binding of short peptides to DNA. It should be emphasized that normally, DNA exists in the form of a double helix, while its transcription and replication requires the separation of the double-helix strands, and that this can also be made to occur by exposure to a temperature of 69.5 °C. Cheng et al., using ethidium bromide substitution and gel electrophoresis, investigated the ability of synthesized cyclic and linear short peptides to bind and cleave the DNA (at temperatures below 60 °C). The authors concluded that the tetrapeptide cyclo[Lys-Trp-Lys-Ahx-] is a promising agent for DNA cleavage as it has better nucleic acid binding and cleavage constants compared with its linear analog and the peptide cyclo[Lys-Tyr-Lys-Ahx-] [77]. 

Another study of DNA in solution demonstrated its separation into separate strands upon the addition of the short peptide AEDG, and that this occurred at a temperature of 28 °C, being characterized by a neutral pH and an approximately two-fold decrease in the entropy and enthalpy indices [78].

The next group of studies used various techniques to analyze the nature of short peptide binding to DNA. Circular dichroism spectroscopy demonstrated that the peptide dimer KGVCV-N2H2Dns2 can interact with oligonucleotides. The probability of such an interaction and the stability of the complexes to increasing NaCl concentrations decrease in the following order: poly dG, poly dC, poly dA, poly dT, poly dGC, poly dGC. The association constant of the peptide-oligonucleotide complex is 20 times higher for poly dG and poly dC sequences than those of poly dA and poly dT [79].

**Figure 2:**
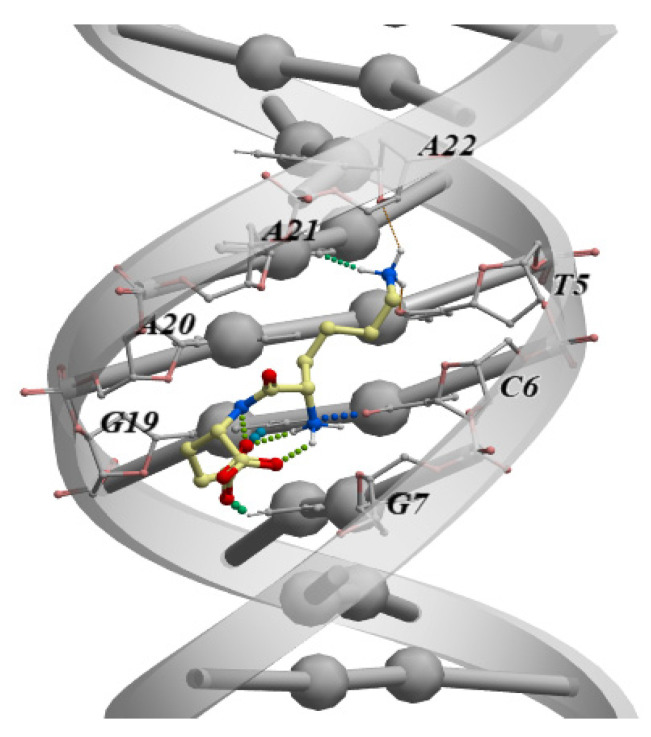
The classical B-shaped binding of the KE dipeptide to the “TCGA” region of the dsDNA. The measurement was performed by molecular modeling and ligand docking using the ICM-Pro software (Molsoft LLC, San Diego, CA, USA).

Another study using molecular modeling methods analyzed the ability of all 400 dipeptide variants (all possible combinations of 20 standard amino acids) to bind all possible combinations of double-stranded DNA tetranucleotides (dsDNA) in the classical B-form. Among all the options, 57 dipeptides were identified with high selectivity of binding to dsDNA [80]. The authors noted that blocking at the ends of the dipeptides with the standard capping groups N-(CH3-CO-NH-) and C-(-CO-NH2) increases the binding performance. It should be emphasized that the immunoprotective peptide KE, previously mentioned in this review, and which is part of the drug Thymalin, showed the best binding parameters to the TCGA dsDNA sequence among all the free-end dipeptides (Figure 2). In an earlier study using molecular dynamics and molecular docking methods, it was also shown that the amino acids included in the KE peptide can additionally interact with dsDNA (Figure 2). However, the sDNA–peptide interaction energy is higher than that with individual amino acids. This allowed the authors to conclude that its peptide bonds enhance the interaction of the KE peptide with dsDNA [81].

Amino acids that carry charges on their side groups can bind to dsDNA and alter the strength of the double helix. Measurement of the melting point of dsDNA (Tm) showed that acidic amino acids (Glu, Asp) weaken hydrogen bonds between the dsDNA strands, while basic amino acids (Arg, Lys) enhance the interactions between strands. There is a rank correlation between the isoelectric points of the amino acids and the Tm changes observed. A similar dependence of the hyperchromic effect on the isoelectric point of proteins (pepsin, insulin, cortexin, and protamine) was found for dsDNA-protein complexes at room temperature. The short peptides KE, AEDG, and KEDP, containing a mixture of acidic and basic amino acid residues, also affect Tm and dsDNA stability. It was found that Glu in its untwisted form binds to the large groove of dsDNA and destroys 3 hydrogen bonds, thus destabilizing the dsDNA. In contrast, Lys in its untwisted form binds to the outer surface of dsDNA and forms two bonds with oxygen atoms of neighboring phosphodiester groups, thus stabilizing the dsDNA structure [82]. 

The interaction of the neuroprotective peptide EDR, which is a component of the drug Cortexin, with dsDNA has been studied by nuclear magnetic resonance, viscometry, and molecular dynamics. It was established that EDR can interact with the large groove of dsDNA, forming bonds with the N7 and O6 atoms of guanine. Mg^2+^ ions enhance the binding of EDR to dsDNA due to their ability to screen the negatively charged phosphate groups of the dsDNA [83]. A similar mechanism of binding to dsDNA was established for AEDL, a bronchoprotective peptide [41]. 

The short peptides AEDG, EDR, AEDL, KEDG, AEDR, and KEDW either inhibit or stimulate hydrolysis of λ bacteriophage DNA by means of WEN1 and WEN2 eukaryotic endonucleases, depending on the state of DNA methylation. Thus, the peptides can not only identify specific DNA sequences, but also their methylation status. In addition to interacting with dsDNA, in eukaryotes, peptides can bind to oligonucleotides containing NG- and CG-sites when methylated [84]. 

The peptide motif SPKK in proteins that regulate gene expression can bind to the minor dsDNA groove. This peptide motif has been linked to the anticancer drug Amsacrine to enhance its interaction with dsDNA. Two bifunctional molecules have been designed and synthesized, where the amsacrine-4-carboxamide derivative was bound to either one or to two SPKK motifs with a 2-aminopropyl core. Both peptide conjugates interact with the major and minor grooves of dsDNA. This process is not impacted by the interaction of the dsDNA with histones [85,86]. However, the probability of the SPKKSPKK octapeptide binding to the minor groove of dsDNA depends on the (non-)availability of histone proteins [87]. The binding of the SPKK peptide motif to intercalating drugs is proving to be an effective system to stabilize drug-DNA complexes. 

It is assumed that short peptides derived from chromosomal proteins specifically bind to the minor dsDNA groove at sites rich in poly A- and poly T-sequences [88]. Binding of the DNA of A- and T-specific dsDNA motifs to the SPRKSPRK peptide has been investigated by using NMR spectroscopy. It was found that the SPRKSPRK peptide nonspecifically interacts with the CGCAAAAAAGGC and GCCTTTTTTGCG dsDNA sequences. In this case, the TPKRPRGRPKK, PRGRPKK, and PRGRP peptides obtained from the HMG-I/Y non-histone chromosomal protein specifically bind to the CGCAAATTTGCG and CGCGAATTCGCG dsDNA sites. The RGR fragment of each peptide contacts the minor groove of the dsDNA along the arginine side chains [89]. The researchers believe that the availability of arginine in short peptides is important for their specific binding to any part of the minor groove of dsDNA that contains poly A and poly T sequences. Another study identified the interaction of a short peptide fragment from the bZIP protein with A/T-rich oligonucleotides. This peptide fragment stabilizes the oligonucleotide structure [90]. 

The literature provides few sources related to attempts to specify the purpose of the peptide-DNA binding sites. Recently, Etzion-Fuchs et al. published a report on a new ensemble machine learning dSPRINT method that offers, among its wide range of objectives, characterization of the molecular functions of the protein domains that bind to nucleic acids, ions, and peptides, as well as the association of these domains with the human genome [91]. Despite the fact that this algorithm is intended for analysis of the interaction of the large protein domain with DNA and other ligands, it is likely that this approach can be adjusted for use with short peptides. 

One cannot rule out the possibility that an identified nucleotide sequence in the DNA, to which a short peptide binds, may belong to the promoter region of genes demonstrating peptide-regulated expression. In this context, attention should be paid to studies in which the specific promoters of human genes, where the DNA sequences to which short peptides bind, have been identified. In a recent study, it was demonstrated, using molecular dynamics methods, that the neuroprotective peptide EDR interacts with a dsDNA sequence that has been identified in the promoter regions of genes involved in the pathogenesis of Alzheimer’s disease (CASP3, TP53, SOD2, GPX1, PPARA, PPARG, NES, GAP43, SUMO1, APOE, and IGF1) [61]. This result allowed the authors to identify the mechanism of neuroprotection by this peptide. 

The study of the properties of the peptide KEDW by means of UV spectroscopy, circular dichroism, and molecular modeling has demonstrated that this tetrapeptide binds to the DNA in association with the sequence ACCT, which is found in the promoter regions of genes responsible for the functional state of pancreatic cells. The authors used this result to present an explanation of the previously identified properties of the tetrapeptide [42].

**Figure 3:**
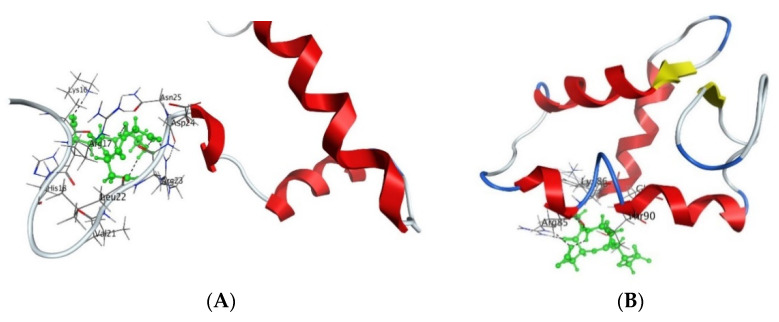
Interaction of AEDG peptide with H4 histone (**A**) and H1/6 histone (**B**) in accordance with the data obtained from the forcefield molecular modeling (Molecular Operating Environment, forcefield Amber12EHT). Histone molecules (Protein Data Bank) are depicted as α-helical domains and loops. Oxygen atoms are shown in red, nitrogen atoms in blue, carbon atoms in black, and hydrogen atoms in light gray. The peptide is highlighted in green. The dotted line shows hydrogen bonds.

The above-mentioned analysis of the binding of dipeptides to dsDNA has detected the specific dsDNA nucleotide sequence TCGA that binds the KE peptide, in the *APG5L* gene promoter (Figure 3).

It should be noted that in an in vitro study, the KE peptide regulated the expression of this gene (Table 2), and that this correlates with the results of the molecular modeling. Similar studies have been conducted for the EW peptide (Table 4). 

Molecular modeling showed that the EW dipeptide binds to dsDNA in the classical B-form according to the presence of the GGAG nucleotide sequence. This nucleotide sequence has been identified in 2 promoters of the *ATP* gene, in 4 promoters of the *MT-CO1* gene, and in the promoter of the *HBA1* and *HSP90* genes, with the expression of all these being regulated by this peptide in in vitro experiments (Table 1 and Table 2) [42]. 

Thus, this review is the first work to trace the inter-relation of physical and chemical, molecular modeling, and studies involving molecular biology. Systematization of the analyzed results has, for the first time ever, allowed identification of the mechanism of the peptide regulation of gene expression at the level of interaction with DNA. The agreement between the above-mentioned studies can be considered as confirmation of the earlier hypothesis that short peptides have a wide range of biological effects by means of DNA–peptide interactions and the regulation of expression of the genes responsible for the functional state of the human body. 

### 4.2. Short Peptides + Histone Proteins

Present-day ideas about the regulation of gene expression are impossible without understanding the epigenetic mechanisms, the main components of which are the histone proteins. Histones are nuclear proteins that provide the packaging of the DNA strands inside the nucleus. Studying the interaction of short peptides with such histone proteins offers a major complement to understanding the mechanisms of their interaction with DNA and the regulation of gene expression.

The ability of short peptides to release genes [92] repressed as a result of the heterochromatinization of euchromatic regions of the chromosomes that occurs during senescence [93] has been demonstrated. In another study, it was shown that amino acids, as well as dipeptides and tripeptides, can interact with the cell chromatin [94].

Analysis of fluorescence quenching showed that the short peptides AEDG, EDR, AEDL, KEDG, AEDR, and KEDW bind to FITC-tagged H1, H2B, H3, and H4 wheat histones (Figure 3) in the region of the peptide-binding motifs of the N-end sections. The authors indicate that peptides with different charges can bind to different motifs: for instance, the amino acid sequence kaakakk serves as the motif for the AEDL and AEDG peptides, whereas evaa has the corresponding function for peptides containing lysine or arginine residues. It should be emphasized that the authors reported that DNA and oligonucleotides in combination with the histones can enhance or prevent peptide binding [15]. 

Molecular modeling has demonstrated that the neuroprotective EDR and DS peptides can bind to the H1.3 histone. The authors assume that such binding can affect the H1.3 histone conformation and lead to a modification of the chromatin structure at the loci of certain genes, in particular, *Fkbp1b*, which encodes the FK506-binding protein. The *Fkbp1b* gene encodes peptidyl-prolyl cis-trans-isomerase that regulates the release of calcium ions from the sarcoplasmic and endoplasmic reticulum of neurons. Activation of *Fkbp1b* gene transcription by EDR or DS can therefore allow the synthesis of its protein product and thus the release of calcium ions from the sarcoplasmic and endoplasmic reticulum of the treated neurons [95]. Another study showed that the peptide AEDG can interact with the H1/6 and H1/3 histones through the His-Pro-Ser-Tyr-Met-Ala-His-Pro-Ala-Arg-Lys and Tyr-Arg-Lys-Thr-Gln amino acid sequences that interact with DNA. The authors associate this property of the peptide with its impact on the expression of neurogenetic genes [17,20]. Thus, the epigenetic mechanism of the peptide regulation of the functional activity of neurons can be traced.

Thus, site-specific interactions of short peptides with histone proteins can serve as epigenetic control mechanisms for chromatin. Recent studies suggest the need for a comprehensive analysis of the interaction of short peptides with DNA and with histones to identify the mechanisms of regulation of gene expression and of the functional activity of cells in the human body. 

## 5. Conclusions

The diverse spectrum of the biological effects of the short peptides that ensure the control of key processes in the body indicates the importance of studies of the mechanisms of their action (Table 5). 

Multiple studies have shown that the main mechanism of action of such peptides is likely to be their ability to regulate gene expression and protein synthesis. This review has focused on a comprehensive analysis of the possible mechanism for the regulation of gene expression being through the interaction of these short peptides with DNA (Table 5).

Short peptides regulate the expression of a wide range of genes related to various types of functional activity of cells and to a diverse range of biological processes: energy homeostasis, cell stress, apoptosis, etc. Moreover, peptides can regulate the physiological activity of cells in the case of senescence. Thus, peptides can be used to support the functional state of cells during the treatment of age-associated diseases. 

The review provided information on the genes and proteins that are involved in the development of such pathologies as immune deficiency, various types of neuropathies, and oncological diseases. It was found that peptides regulate the expression of genes involved in the pathogenesis of specific diseases. This indicates the promising prospects for the use of short peptides as physiologically active components of drugs. 

An important aspect in studying the mechanism of peptide action has been analysis of the binding of dipeptides to DNA, which has contributed significantly to the identification of specific DNA. These sequences can be found in the promoter regions of genes, the expression of which is regulated by these short peptides. Molecular modeling as well as physical and chemical methods established the binding of short peptides to histones, resulting in potential for modification of the chromatin structure. 

This systematic review has revealed the similarity of the results of multiple studies conducted with the use of molecular biology, physical and chemical methods, and bioinformatics. It is beyond argument that further research is required to expand our understanding of the molecular genetic mechanisms of peptide regulation.

However, even at this stage, we can confidently predict that there is a common mechanism for the peptide regulation of gene expression and protein synthesis dependent upon DNA–peptide interactions.

## Figures and Tables

**Table 1:** Genes and proteins involved in the pathogenesis of various diseases, the expression of which is regulated by the EW peptide.

Gene	Protein	Function/Biological Process	Disorders
*MT-ATP6 (ATP6)*	ATP synthase subunit a	ATP synthesis, hydrogen ion transport	Neuropathy, ataxia, retinitis pigmentosa, Leigh Syndrome
*MT-ND1*	NADH-ubiquinone oxidoreductase core subunit 1	electron transport	Leber hereditary optic atrophy, mitochondrial complex I deficiency, MELAS syndrome, Leber hereditary optic neuropathy, dystonia
*MT-ND4*	NADH-ubiquinone oxidoreductase chain 4		Leber hereditary optic neuropathy, modifier of mitochondrial myopathy, encephalopathy, lactic acidosis, stroke-like episodes
*MT-CO1*	cytochrome c oxidase subunit 1		Deafness, non-syndromic sensorineural, mitochondrial, and genetic recurrent myoglobinuria
*AK2*	adenylate kinase 2	cellular energy homeostasis, adenine nucleotide metabolism	Reticular dysgenesis, immunoerythromyeloid hypoplasia, atopic dermatitis, severe combined immunodeficiency
*HBA1,2*	Hemoglobin subunit alpha	oxygen transport	Erythrocytosis, familial 7 and hemoglobin h disease, Alpha-thalassemia
*COP1*	E3 ubiquitin-protein ligase COP1	ubiquitination and proteasomal degradation of target proteins	autism
*PDLIM5 (Enh2)*	PDZ and LIM domain protein 5	regulation of cardiomyocyte expansion, heart development by scaffolding PKC to the Z-disk region	Nail-Patella syndrome, bipolar disorder
*HSP90AB1*	heat shock protein 90 family	protein folding and degradation, gastric apoptosis, and inflammation	Larynx cancer, Powassan encephalitis
*HSPBAP1 (Pass1)*	27 KDa Heat Shock Protein-Associated Protein 1	cellular stress response, cell growth and differentiation	Renal cell carcinoma, nonpapillary, epithelial recurrent erosion dystrophy
*гeны HLA*	human leucocyte antigens	MHC class II receptor activity/adaptive immunity, host–virus interaction, innate immunity	Rheumatoid arthritis, type 1 diabetes mellitus, cardiac sarcoidosis, measles, berylliosis, granulomatosis with polyangiitis, Halo Nevi, polyarticular juvenile idiopathic arthritis, pityriasis rosea, fetal and neonatal alloimmune thrombocytopenia, Graham-Little-Piccardi-Lassueur syndrome, penicillin allergy, human cytomegalovirus infection, asthma, severe pre-eclampsia, celiac disease 1, adult-onset myasthenia gravis, psoriasis 1, human immunodeficiency virus type 1, severe cutaneous adverse reaction, birdshot chorioretinopathy, celiac disease 1, Creutzfeldt-Jakob disease, sarcoidosis 1, multiple sclerosis

**Table 2:** Genes and proteins involved in the pathogenesis of various diseases, the expression of which is regulated by the KE peptide.

Gene	Protein	Function/Biological Process	Disorders
*EPS15*	epidermal growth factor receptor pathway substrate 15	clathrin-mediated endocytosis and development of HGF signaling pathway.	Vaccinia, cataract 8 multiple types, Menkes disease, autosomal recessive spastic paraplegia type 20
*MCM10*	minichromosome Maintenance 10 Replication Initiation Factor	cell proliferation, cellular response to DNA damage, DNA replication	Immunodeficiency 80 with or without congenital cardiomyopathy, Baller-Gerold syndrome, Rapadilino syndrome, Rothmund-Thomson syndrome type 2, Fanconi anemia
*Cul 5*	cullin-5 (CUL-5, vasopressin-activated calcium-mobilizing receptor 1, VACM-1)	core component of multiple SCF-like ECS (Elongin-Cullin 2/5-SOCS-box protein) E3 ubiquitin-protein ligase complexes, which mediate the ubiquitination and subsequent proteasomal degradation of target proteins.	Molluscum contagiosum, Cockayne syndrome, lung cancer
*APG5L*	autophagy protein 5	autophagic vesicle formation, mitochondrial quality control after oxidative damage, negative regulation of the innate antiviral immune response, lymphocyte development and proliferation, MHC II antigen presentation, adipocyte differentiation, apoptosis	Spinocerebellar ataxia 25, stomatitis
*ZNF01*	zinc finger protein 1 homolog	nucleic acid binding, DNA-binding transcription factor activity	Retinoblastoma and neuropathy
*FLJ12848 fis (TNPO3)*	transportin-3	nuclear import signal receptor activity, small GTPase binding	Muscular dystrophy, limb-girdle, autosomal dominant 2
*ITPK1*	inositol-tetrakisphosphate 1-kinase	inositol phosphate metabolism, necroptotic process, neural tube development	Neural tube defects
*SLC7A6*	Y + L amino acid transporter 2	amino acid transmembrane transport, leukocyte migration, ornithine transport	Lysinuric protein intolerance, hepatocellular carcinoma cystinuria, persistent fetal circulation syndrome
*KIAA0699 (BICD2)*	protein bicaudal D homolog 2	Golgi-to-ER retrograde transport	Spinal muscular atrophy, lower extremity-predominant, autosomal dominant
*FLJ10914 (MRGBP)*	MRG/MORF4L-binding protein	acetylation of nucleosomal histones H4 and H2A	Colorectal cancer, colorectal adenoma
*Gdap1*	Ganglioside-induced differentiation-associated protein 1	glutathione metabolic process, mitochondrial fission, mitochondrial fusion, protein import into peroxisome membrane, protein targeting to mitochondrion	Charcot-Marie-Tooth disease
*MSTP028 (KCTD10)*	BTB/POZ domain-containing adapter for CUL3-mediated RhoA degradation protein 3	DNA synthesis and cell proliferation	CBlB type of methylmalonic aciduria, occupational dermatitis

**Table 3:** Genes and proteins involved in the pathogenesis of various diseases, the expression of which is regulated by the AEDG peptide.

Gene	Protein	Function/Biological Process	Disorders
*RAD21*	Double-strand-break repair protein rad21 homolog	apoptosis, cell cycle, cell division, chromosome partition, DNA damage, DNA repair, mitosis, transcription, transcription regulation	Cornelia de Lange syndrome 4, Mungan syndrome
*TOP3B*	DNA Topoisomerase III Beta	DNA recombination, cellular aging, and maintenance of genome stability	Chromosome 22Q11.2 Duplication Syndrome Prosopagnosia
*AK2*	Adenylate kinase 2	cellular energy homeostasis, adenine nucleotide metabolism	Reticular dysgenesis, immunoerythromyeloid hypoplasia, atopic dermatitis, severe combined immunodeficiency

**Table 4:** Human gene promoters in which the nucleotide sequence of dsDNA, to which the EW peptide binds, has been found.

Gene Promoters	Nucleotide Sequence
1 *ATP* promoter	GGGCGGGGGCAACGGTCACCTGATCTGCGGCTGTCGAGGCCGCTGAGGCAGT**GGAG**GCTG
2 *ATP* promoter	CAGCTGTCCCAGCGGAAGCGACGAAGGGACGGGACCCG**GGAG**CCTGGACGAGTCCGAGCG
1 *MT-CO1* promoter	CGGGC**GGAG**TCTTCCTCGATCCCGTGGTGCTCCGCGGCGCGGCCTTGCTCTCTTCCGGTC;
2 *MT-CO1* promoter	CATTAACGGGAACAAATTCTCTTTACACAAAGCTCAGGCACATTCAATCAAGG**GGAG**CCA
3 *MT-CO1* promoter	GCCCCCGCCCGCTCC**GGAG**CAACCCGCGAGCTTACACCGGCTTCTCTCTGTCCTCAGCCC
4 *MT-CO1* promoter	GTGATTGGCCCAGAGAGG**GGAG**GTGACCCCAGGCCCCAGGAAAG**GGAG**CGAGGACAGCGC
*HBA1* promoter	GAGTATGGTGC**GGAG**GCCCT**GGAG**AGGTGAGGCTCCCTCCCCTGCTCCGACCCGGGCTCC
*HSP90* promoter	TTCCAGATGCCTGAGGAAACCCAGACCCAAGACCAACCGAT**GGAG**GA**GGAG**GAGGTTGAG

From hsEPDnew, the Homo sapiens (human) curated promoter database (https://epd.epfl.ch/human/human_database.php?db=human, accessed on 1 October 2019). The sequences of nucleotides with which the peptide binds are highlighted in red.

**Table 5:** Biological effects of the short peptides.

N	Structure and Name of Peptide	Biological Activity	References
	Polyfunctional Peptides		
1	AED, Cartalax	regulation of cartilage and skin fibroblasts functions, neuronal cell differentiation	[17,18,21,34,35,74]
2	AEDG, Epitalon	regulation of neuro-immuno-endocrine function, circadian rhythm regulation, retina-protective effect, antioxidant effect, stress-protective effect, geroprotection, activation of skin fibroblasts’ function, differentiation of plant cells, DNA binder	[15,16,19,21,23,34,40,51,78,82,84]
3	AEDL, Bronchogen	regulation of lung cells’ function and differentiation, differentiation of plant cells, DNA binding	[15,16,24,35,41,84]
4	EDL, Ovagen	regulation of renal cells’ function, hepatoprotection, DNA binding	[74]
5	EDR, Pinealon	neuroprotection, activation of stem cells’ neuronal differentiation, antioxidant effect, DNA binding	[15,61,83,84,95]
6	EW, Thymogen	drug, regulation of immune system function, antioxidant effect, stress-protective effect, geroprotection, DNA binding	[23,42]
7	KE, Vilon	regulation of immune system function, antioxidant effect, stress-protective effect, geroprotection, activation of stem cells’ neuronal differentiation, activation of plant cells’ differentiation, DNA binding	[16,17,18,21,23,34,35,81,82]
8	KED, Vesugen	regulation of cardiovascular system function, neuroprotector, activation of stem cells’ neuronal differentiation, activation of skin fibroblasts’ function, geroprotection, DNA binding	[17,18,21,22,34,35,60,75]
9	KLDL	osteogenic and chondrogenic differentiation of stem cells	[37]
10	RADA	osteogenic and chondrogenic differentiation of stem cells	[37]
	**Monofunctional peptides**		
11	AEDR, Cardiogen	regulation of cardiovascular system function	[15,84]
12	RADA in combination with the Jagged1	heart progenitor cell differentiation	[37]
13	KEDG, Testagen	regulation of male reproductive system function	[15,34,35]
14	AAAAEKAAAAEKAAAAEK	neuroprotection	[35,40]
15	MEHFPGP, Semax	drug, neuroprotection	[53,54,55,56,57]
16	TKPRPGP	neuroprotection	[58,59]
17	IKVAV	stem cells’ neuronal differentiation	[36]
18	IRW	osteogenic differentiation of stem cells	[35,43]
19	GRGDS	osteogenic differentiation of stem cells	[35,44,45]
20	YCWSQYLCY	osteogenic differentiation of stem cells	[46]
21	AcSDKP	activation of skin fibroblasts’ function	[75]
22	TKPRPGP	immunoprotection	[58,59]
23	Ac-SHAVSS-NH2, HAV	regulation of the gene expression involved in E-cadherin synthesis	[52]
	**Peptides with unknown biological function**		
24	cyclo[Lys-Trp-Lys-Ahx-]	DNA binding	[77]
25	peptide dimer KGVCV-N2H2Dns2	DNA binding	[79]
26	PRGRP	DNA binding	[89]
27	PRGRPKK	DNA binding	[89]
28	RGR	DNA binding	[89]
29	SPKK	DNA binding	[85,86,87]
30	SPRKSPRK	DNA binding	[89]
31	TPKRPRGRPKK	DNA binding	[89]

## Data Availability

Not applicable.
